# Children’s Preferences for Oral Dosage Forms and Their Involvement in Formulation Research via EPTRI (European Paediatric Translational Research Infrastructure)

**DOI:** 10.3390/pharmaceutics13050730

**Published:** 2021-05-15

**Authors:** Elisa Alessandrini, Francis Brako, Mariagiovanna Scarpa, Mariangela Lupo, Donato Bonifazi, Valeria Pignataro, Maria Cavallo, Ornela Cullufe, Cristina Enache, Begonya Nafria, Joana Claverol, Leen De Taeye, Eric Vermeulen, Jennifer Preston, Catherine Tuleu

**Affiliations:** 1 Department of Pharmaceutics, University College London School of Pharmacy, 29-39 Brunswick Square, London WC1N 1AX, UK; f.brako@kent.ac.uk (F.B.); m.scarpa@alumni.ucl.ac.uk (M.S.); c.tuleu@ucl.ac.uk (C.T.); 2 Medway School of Pharmacy, Universities at Medway, Anson Building, Chatham ME4 4TB, UK; 3 TEDDY European Network of Excellence for Paediatric Research, via Luigi Porta 14, 27100 Pavia, Italy; mlupo@teddynetwork.net; 4 Consorzio per Valutazioni Biologiche e Farmacologiche, CVBF, via N. Putignani 178, 70122 Bari, Italy; coordinator@eptri.eu (D.B.); vpignataro@cvbf.net (V.P.); mcavallo@cvbf.net (M.C.); 5 Consorzio per Valutazioni Biologiche e Farmacologiche, Dege e Shoqerise se Huaj, CVBF Albania, Rr.Prokop Myzeqari, 1000 Tirana, Albania; ocullufe@cvbf.net; 6 The Romanian Angel Appeal Foundation, Strada Rodiei, 030956 București, Romania; cristina.enache@raa.ro; 7 Sant Joan de Déu Research Institute, Esplugues de Llobregat, 08950 Barcelona, Spain; bnafria@sjdhospitalbarcelona.org (B.N.); jclaverol@fsjd.org (J.C.); 8 Department of Internal Medicine and Paediatrics, Ghent University, 9000 Ghent, Belgium; Leen.DeTaeye@ugent.be; 9 Dutch Patient Alliance for Rare and Genetic Diseases, VSOP, Koninginnelaan 23, 3762 DA Soest, The Netherlands; e.vermeulen@vsop.nl; 10 Department of Women’s and Children’s Health, Institute of Life Course and Medical Sciences, Alder Hey Children’s NHS Foundation Trust, Eaton Rd, Liverpool, L12 2AP, UK; Jennifer.Preston@liverpool.ac.uk

**Keywords:** paediatric formulations, oral dosage forms, medicines acceptability, EPTRI, patient-centric research

## Abstract

The paucity of evidence-based data on formulation characteristics preferred by the children is known to limit the design of tailored paediatric dosage forms. The European Paediatric Translational Research Infrastructure (EPTRI) commissioned a study to evaluate children’s dosage forms perceived preferences in some European countries and explore the feasibility of using the young persons advisory groups (YPAGs) to involve children in formulation research. An online, age-adapted survey was developed and translated into six languages. The survey link was disseminated across seven European countries: Albania, Italy, the Netherlands, and Dutch-speaking part of Belgium, Romania, Spain, and the United Kingdom. Respondents’ (*n* = 1172) perceived preferences for oral dosage forms primarily differed based on age, health status, and experience. Conventional dosage forms, i.e., liquid (35%), tablets (19%), and capsules (14%), were the most selected. Liquid was widely selected by children less than 12 years and by those healthy and taking medicines rarely. Monolithic solid forms were mostly chosen by adolescents and by children with a chronic disease taking medicines frequently. There was a clear lack of familiarity with more novel dosage forms (e.g., orodispersible films and granules). Noteworthy, granules were not appreciated, particularly by adolescents (52.8%). To rationalise the creation of paediatric formulations, it is important to involve children as active stakeholders and to apply tools assessing children’s perspectives on medicines to inform acceptable dosage form development from the start.

## 1. Introduction

Nowadays, increasing efforts and resources are dedicated to paediatric medicine development to guarantee the authorisation and access to tailored and high-quality medicines for children. One of the barriers slowing the development of age-appropriate paediatric medicines is the lack of knowledge about what dosage forms are considered to be acceptable to the paediatric population [1].

Acceptability is defined as “the overall ability and willingness of the patient to use and its caregiver to administer the medicine as intended” [2,3,4]. This requirement is paramount to guarantee optimal treatment adherence and consequently the efficacy and safety of a therapy [3,4]. Thus, studying the acceptability of paediatric formulations has now become of great importance, and the evaluation of patient acceptability has become an integral part of the pharmaceutical and clinical development of paediatric medicines. Therefore, pharmaceutical companies in Europe and the United States are now obliged to describe and justify in their paediatric development plans (PIP and PSP) the choice of their formulations for all target populations and to document and report the acceptability of them [1,5]. However, there is still a well-acknowledged paucity of evidence-based data to help define key dosage form characteristics likely to be age-appropriate and acceptable across this very heterogeneous population [2,6,7]. This creates several challenges for formulation scientists to propose suitable preparations [2].

In the last years, the idea that children’s views and preferences should be taken into account has spread across pharmaceutical organisations, regulators, and the public. For this reason, healthcare professionals have begun to consider the active participation of patients and families as a fundamental step to reach consensus and compliance to treatments and involvement in research [3]. Young persons’ advisory groups (YPAGs) have become popular within academic institutions. YPAGs are an example of patient and public involvement (PPI), allowing young individuals from the patient and general population to have a voice in scientific research [8]. They are groups of children and adolescents usually involved in the design and delivery of clinical research as active and reflective participants, and their direct engagement in research is considered favourable to both researchers and children [9]. However, very few PPI activities currently focus on basic research, as they usually look at the clinical research part.

Asking children their opinions and preferences about oral dosage forms during the formulation research phase is key for planning and developing better, child-appropriate dosage forms, and it reflects the evolvement of clinical pharmaceutics towards true patient-centred formulation development [10,11]. This approach would allow a better understanding of the range of factors affecting children’s medicines preferences, acceptability, and ultimately treatment outcomes.

In this regard, the European funded project European Paediatric Translational Research Infrastructure (EPTRI) commissioned a case study aimed at collecting information about children and adolescents’ experiences and preferences for oral dosage forms across various European countries by using an online child-friendly survey developed in collaboration with YPAGs.

EPTRI arises from the serious lack of medicines for children. It aims to enhance technology-driven research in paediatric drug discovery and early development phases to be translated into clinical research and medical practice. In addition, it aims to strengthen collaborations within the scientific paediatric community throughout Europe, to cover the existing gaps in paediatric medicine research. One of EPTRI’s objectives is to federate the necessary expertise and competencies within the pharmaceutical fields to support the development of tailored and accepted paediatric medicines and to facilitate their evaluation in this population. To do so, it aims at establishing a future model of a formulation-research platform to expedite the adoption of innovative technologies for better and safer dosage forms for children. EPTRI is also expected to positively affect the social and ethical aspects of research by involving paediatric patients’ representatives and YPAGs in its advisory bodies to include their point of view in all the activities of the future paediatric research infrastructure to generate a patient-centric approach in the conduct of basic research.

The aim of this study was to look at perceived preferences for oral dosage forms in a large and heterogeneous paediatric population in terms of age groups and countries involved in order to gain further insight into children’s views on oral dosage forms in a real-life setting. Moreover, this study aimed at exploring the feasibility and value of involving the EPTRI PPI group and YPAG advisory bodies in this formulation research activity by seeking their feedback during the development of the online survey. This was important to assess that the survey was age-appropriate and compatible with children’s understanding and abilities. Moreover, the EPTRI PPI and YPAGs helped with the dissemination of the survey.

A previous study conducted by Ranmal et al. [2] also looked at end-users’ perceptions and preferences for oral formulations in children. However, their study was restricted to the UK population aged between 6 and 18 years, with a smaller subgroup study conducted in Montreal, Canada, and they only focused on solid dosage forms administered orally. Our study covered all paediatric age groups across seven European countries and also included liquid dosage forms. Furthermore, the study by Ranmal et al. required direct interaction with the children, firstly because the questionnaire was distributed in laminated copies and secondly because the children were presented with physical models of tablets and capsules to assess what sizes were deemed acceptable. On the contrary, our study aimed to assess the feasibility of using an online survey to reach out to end-users. Thus, it was conducted without any physical interaction with the children.

The availability of these data may help formulation scientists to increase their knowledge about general children’s attitudes towards oral medicines to keep in mind for the development of tailored and acceptable dosage forms for the entire paediatric population.

## 2. Materials and Methods

### 2.1. Study Design

This study was conducted through an online survey distributed to children and their parents in the following countries: United Kingdom, Italy, Spain, Albania, Romania, the Netherlands, Dutch-speaking part of Belgium, Czech Republic, and Sweden. The survey was uploaded on Qualtrics^XM®^ (Provo, UT, USA), an online survey platform used for data collection and analysis. In each country, the survey was disseminated as a web link through several channels: social media, websites, newsletters, YPAG groups, schools, patients’ associations, and research centres, as indicated in Table 1. Across countries, the survey became accessible at different times, whereas the closure date was the same.

Children able to read were encouraged to answer the survey by themselves, while parents or carers of younger children or those unable to complete the survey on their own were asked to help the child. To allow the child to take part in the survey, an adult had to provide their consent. The survey required approximately 5–10 min to be completed.

All data were anonymous and handled and stored in compliance with the General Data Protection Regulation (EU) 2016/679 (GDPR). The survey was sent to the EPTRI Ethics Board for review, which declared that no ethics committee approval was required as the questionnaire only dealt with anonymous data.

### 2.2. Survey Design

The survey was originally developed by the University College London School of Pharmacy in English.

Then a pilot phase involving the EPTRI PPI team and YPAGs members from Italy, Spain, and Albania was conducted between October and December 2018. This phase comprised the revision of the English survey to make sure the questions and language were age-adapted and design and layouts child-friendly. Moreover, during this phase, YPAG members were involved in the translation of the survey into Italian, Spanish, and Albanian. Overall, the survey was considered acceptable, and the feedbacks were discussed during a meeting, where only minor changes were suggested.

After its refinement, the survey was translated into three more languages: Romanian, Dutch, and Czech, and then disseminated in the following nine countries: Italy, Spain, Albania, Romania, United Kingdom, Czech Republic, Sweden, Netherlands, and Dutch-speaking part of Belgium. One single link was provided to respondents of the Netherlands and Dutch-speaking part of Belgium; thus, results have been analysed together as a single country.

The survey was composed of 30 questions, divided into the following sections: background information about the adult helping the child to complete the survey; background information of the child: age, sex, ethnicity, nationality; if the child had or not a chronic health condition; perceived preferences for oral dosage forms: their most and least favourite medicine; rationale for such choices; if the child had already taken the selected dosage forms. (Complete list of questions in the Supplementary Materials). Animated images showing various oral dosage form options were used to make the survey more engaging and easier for the children to make their selections. 

### 2.3. Inclusion Criteria

The main inclusion criteria for selecting participants were age (0 to 18 years) and residency in a designated country included in the study.

### 2.4. Data Extraction and Analysis

Data extraction was performed on the 1st of April 2020. Participants’ responses collected until that date were downloaded from Qualtrics. Data from surveys containing responses in progress but not submitted were excluded from subsequent analysis; also, data from Sweden and the Czech Republic were excluded due to the low response rate at the time of data extraction.

The total number of submitted questionnaires was 2056. Out of these, 884 were excluded because either the adult did not authorise the child to complete the survey and hence automatically terminated, or the survey was not completed. Thus, the analysis was performed on data from 1172 completed surveys, as shown in Table 2.

Responses from countries other than the UK were translated into English to allow analysis and comparisons; then, the data from each of the seven countries were pooled together for analysis. Descriptive and statistical analyses were performed using Microsoft Excel 2016 and GraphPad Prism 8 software. Chi-square tests were performed to compare categorical variables, Spearman’s rank-order correlation to evaluate the relationship between variables, and z-tests to compare two population proportions. A *p*-value significance threshold of 0.05 was used, and the Bonferroni correction was applied where multiple analyses were conducted.

## 3. Results

Of the 1172 participants who completed the online survey, 407 (34.7%) were from Italy, 267 (22.8%) from Spain, 172 (14.7%) from Albania, 135 (11.5%) from Romania, 102 (8.7%) from the Netherlands or Dutch-speaking part of Belgium, and 89 (7.6%) were from the United Kingdom.

Of the questionnaires, 56% were completed by a child alone, 17% with a little help from an adult, and the remaining 27% with a lot of help from an adult.

The adult helping the child to complete the survey was a female 77% of the time, and generally a parent (73%), a sibling (10%), another relative (15%), or a grandparent (1%). The age distribution of the adults was as follows: 22% between 18 and 25 years, 43% between 26 and 44 years, 34% between 45 and 59 years, and 22% were above 60 years old. Most of the adults (66%) had a higher education level (e.g., university degree) or went to a secondary school (29%), while the remaining 5% had a primary school education level or had no education.

The average age of the population surveyed was 12.3 years ± 4.8 standard deviation (SD). After grouping data in three age ranges, more than half of participants (55.4%) belonged to the age group 13–18 years, 28.9% to the age group 7–12 years, and 15.7% to the age group 0–6 years. The age classification adopted in this study differed from that suggested by the ICH harmonised guideline [12]. This because the number of infants and toddlers (between 0 and 23 months) who took part in the study was only 1.5% of the entire population surveyed, and it would not have allowed a fair comparison with the other age groups. This little representation of infants and toddlers was partly due to the types of channels used to disseminate the survey and to the nature of the survey that promoted the direct participation of the child and so might have discouraged parents of young children from taking part.

**Figure 1:**
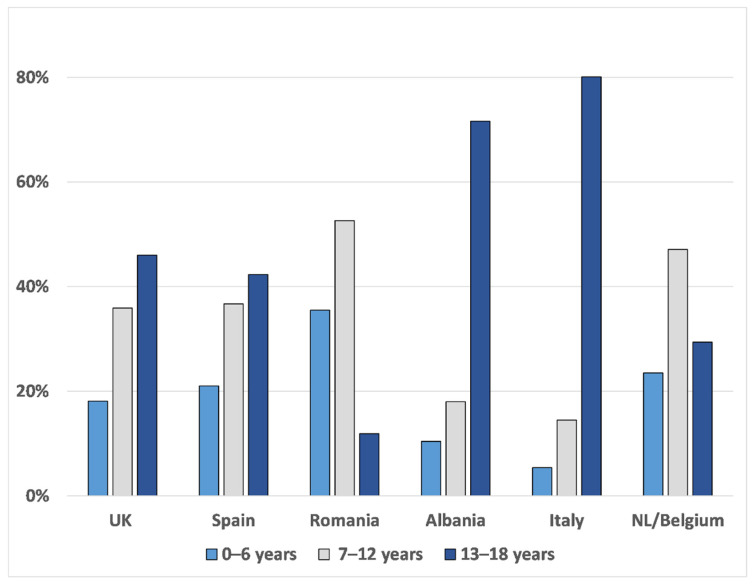
Age distribution of participants (%) per country. Light-blue columns for participants between 0 and 6 years, grey columns for 7–12 years, and blue columns for 13–18 years.

The age distribution of participants per country is represented in Figure 1. Participants from Romania were generally younger than the other countries (mean 8 ± 4.2 years SD), the Dutch followed with an average age of the population of 10 ± 4.5 years SD, whereas in Italy and Albania, more adolescents took the survey. The average age was 14.5 ± 3.7 years SD for Italy and 13.8 ± 4.6 years SD for Albania.

In each country, the proportion of girls and boys taking the survey was similar. The only exception was for Albania, where the percentage of girls was significantly higher (double) that of boys (67.4% vs. 32.6%, *p* < 0.0001).

The majority of respondents were healthy (83%), except in the Netherlands and Belgium, where a slightly higher percentage of children with a chronic health condition took part. The difference was however not statistically significant (56.9% vs. 43.1%, *p* = 0.1657). Table 3 reports an overview of participants’ demographics.

Overall, the most favourite dosage form was liquid (35%), followed by tablets (19%), capsules (14%), effervescent tablets (9%), minitablets (9%), orodispersible tablets (8%), other medicines (3%), orodispersible films and granules (1% each).

**Figure 2:**
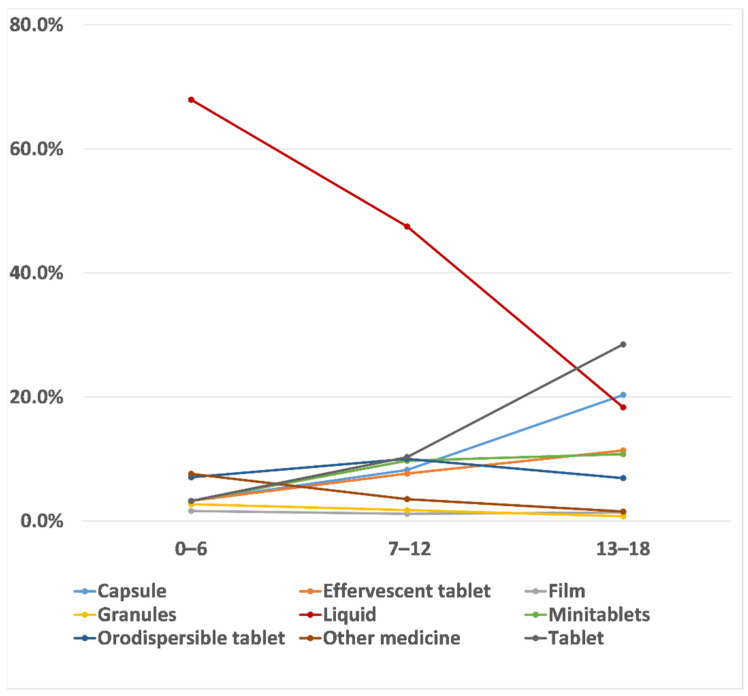
Trends of dosage forms’ perceived preferences among the three age groups (0–6 years, 7–12 years, and 13–18 years) and across all the countries surveyed. Each line represents a dosage form as indicated in the legend.

Considering the great age difference existing in the population studied, an analysis by age groups was performed to appreciate similarities and differences among paediatric subsets. Liquid appeared to be the oral dosage form of choice by the youngest children (67.9% age groups 0–6 years). Its trend steadily declined as age increased (47.5% age group 7–12 years, and 18.3% age group 13–18 years), and this association was statistically significant (Spearman’s rank coefficient value R = −1.28). Contrarily, the selection of tablets and capsules increased proportionally and significantly with the age of the child (R = 0.91 for tablets and R = 0.93 for capsules). Tablets and capsules became the preferred choice for the age group 13–18 years, as shown in Figure 2.

**Figure 3:**
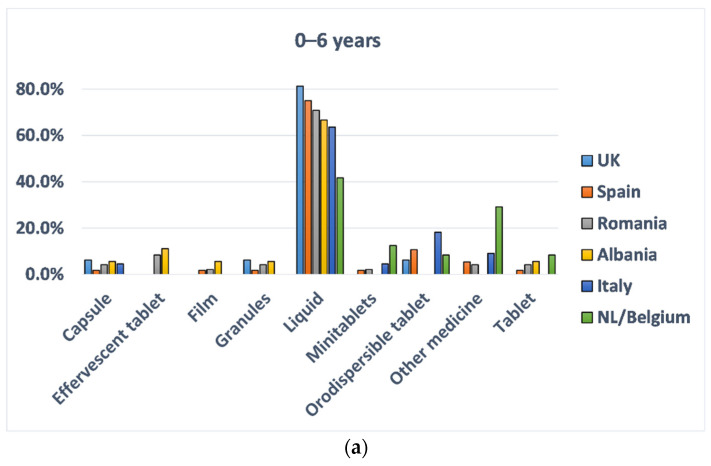

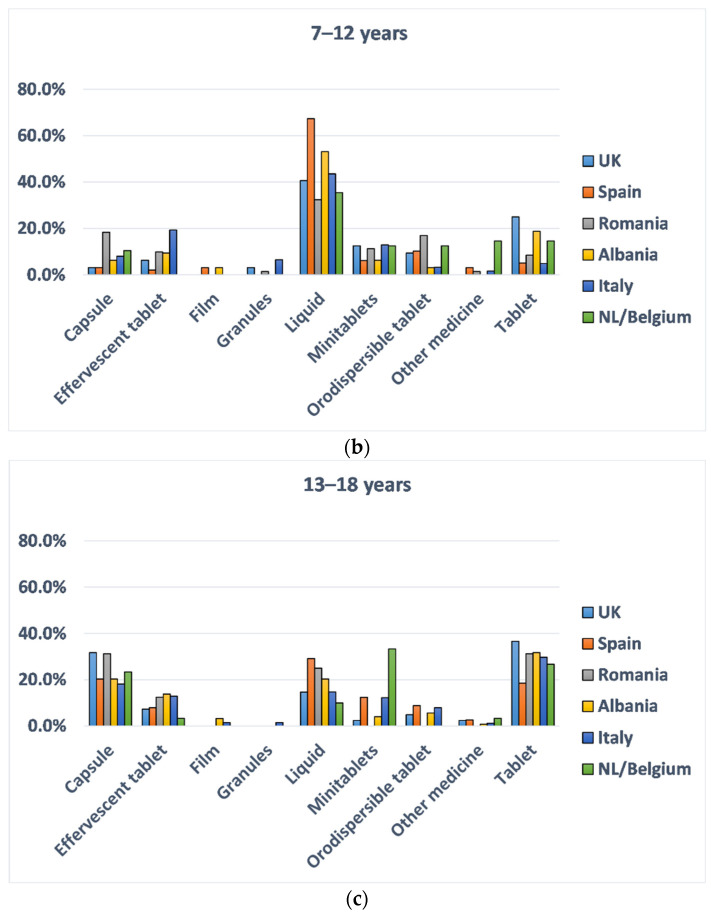
Percentage of respondents by country and type of dosage forms preferred; for (**a**) 0–6 years, (**b**) 7–12 years, and (**c**) 13–18 years. Different colours of the columns indicate different countries, as reported in the legend.

The analysis by countries showed that liquid dosage forms were the favourite choice of all the youngest groups (0–6 years), as reported in Figure 3. In most of the countries, liquid medicines seemed to be almost the exclusive choice in this age group. Of note, 18.2% of Italian participants in the age group 0–6 years selected orodispersible tablets as their favourite choice. Among the Dutch participants, 12.5% selected minitablets, and 29.2% indicated other medicines, which mainly included crushed tablets or the powder from capsules. More variations in choice selections across various countries emerged in the age group 7–12 years, despite liquid still being the first option. For instance, the liquid formulation was still chosen by 67% of Spanish participants. The second preferred dosage forms were: tablets (25%) for the UK and Albania (18.8%), capsules (18.3%) and orodispersible tablets (16.9%) for Romania, effervescent tablets (19.4%) for Italy, and tablets (14.6%) and other medicines (14.6%), including chewable tablets for the Dutch participants. For respondents belonging to the age group 13–18 years, tablets were the most preferred dosage form, and they were the first selected choice by participants of the UK (36.6%), Romania (31.3%), Albania (31.7%), and Italy (29.8%). In addition, capsules were preferred over liquid dosage forms by most of the respondents except for the Spanish and Albanian. In Spain, liquid remained the first favourite dosage form (29.2%). While in Albania, liquid dosage form and capsules were equally preferred after tablets. Interestingly, in the Netherlands and Belgium, the most preferred dosage form for this age group was minitablets (33.3%).

**Figure 4:**
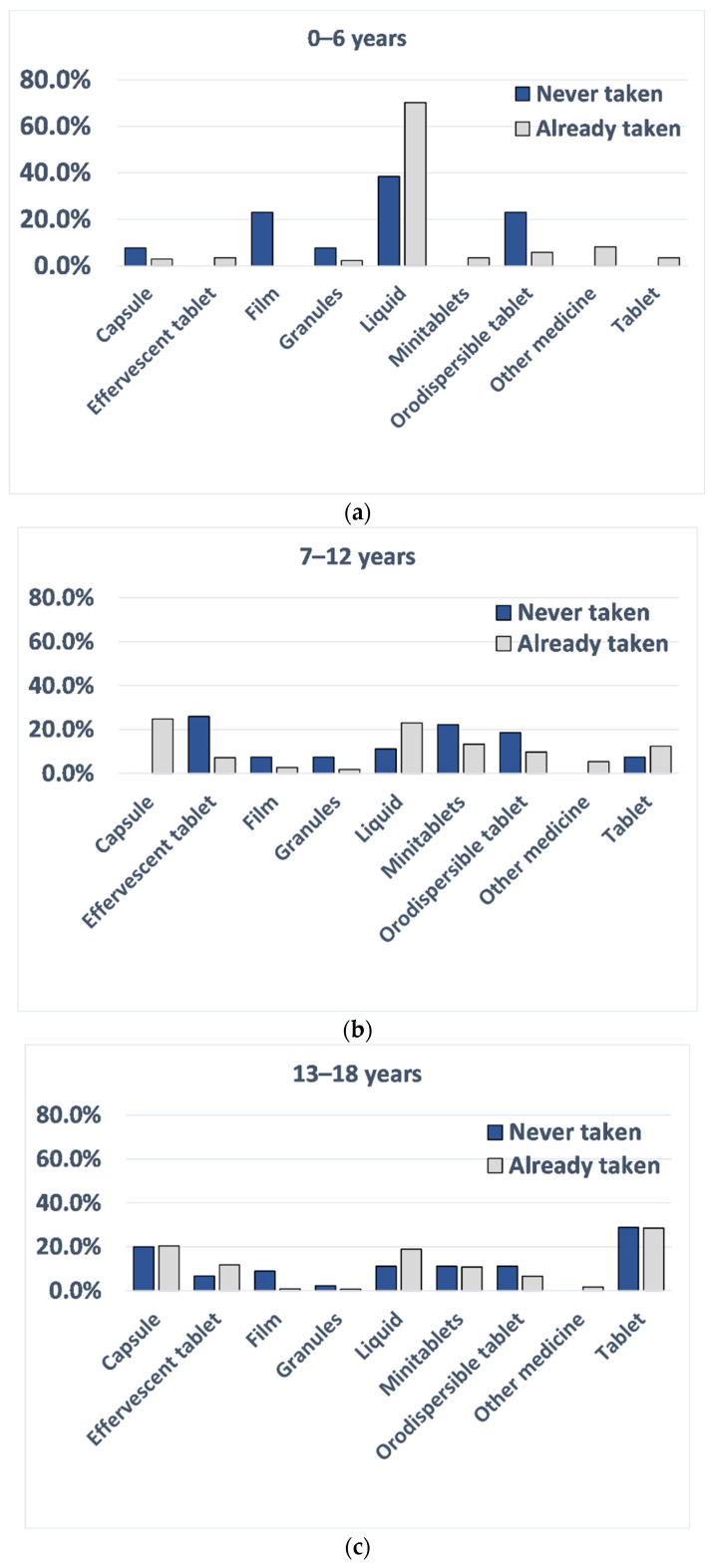
Percentage of respondents and their favourite type of dosage form by previous usage (never taken it, blue columns, already taken, grey columns) for (**a**) 0–6 years, (**b**) 7–12 years, (**c**) 13–18 years.

Children’s preferences were further investigated by those who chose a dosage form that they had already used and those who selected a dosage form never taken before. This was to investigate if choices based on actual experience differed from those based on perception within the same age groups and to understand the value of using data on perceived preferences to predict acceptability and thus determine how reliable such data could be. More than 90% of the participants chose their favourite dosage form based on their current or past experience. When looking at each age group, there seemed to be differences between choices based on experience and those based on perception for children between 0 and 12 years, as shown in Figure 4. In particular, for the age group 0–6 years, liquid was the dosage form largely chosen by those that already used it, whereas orodispersible films and orodispersible tablets were the second and third most selected dosage forms of those who based their selection on dosage forms never used. For the age group 7–12 years, the three most selected dosage forms based on perception (children who never took them before) were: effervescent tablets, minitablets, and orodispersible tablets, suggesting that, although not frequently used, children/parents of this age group had a positive attitude toward these dosage forms. Contrarily, children of the same age, who based their choice on previous experience, selected capsules and liquid dosage forms. Little differences were observed in the age group 13–18 years.

The health status of the children had a great influence over medicine perceived preferences, and there was a statistically significant difference in dosage form selection between children with and without a chronic health condition (*p* < 0.0001). Healthy children, who were taking medicines from time to time, were more inclined to choose medicines easy to swallow, such as liquid or effervescent tablets over solid dosage forms. In contrast, children affected by a chronic condition who were taking one or more medicines daily seemed to prefer solid dosage forms, e.g., tablets or capsules over liquid.

The proportion of children taking medicines from time to time and selecting liquid forms was significantly higher than those taking medicines daily (37% vs. 21%, *p* = 0.0004). On the contrary, the proportion of children taking several medicines every day and selecting tablets was higher compared to those taking medicines rarely (30% vs. 18%, *p* = 0.0145); similarly, the proportion of those selecting capsules and taking medicines once daily was significantly higher (25% vs. 13%, *p* = 0.0059) compared to children taking medicines less frequently.

**Figure 5:**
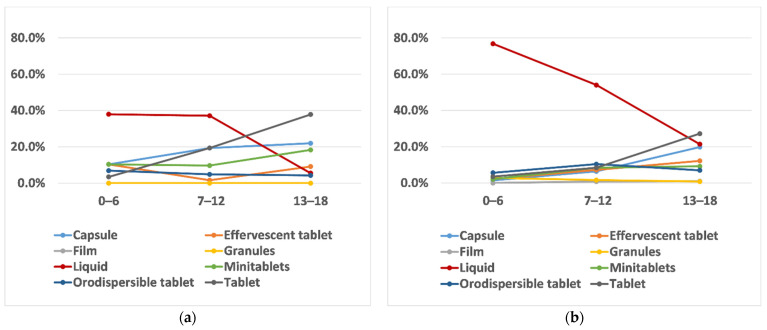
Trends of dosage forms’ perceived preferences among children who had already used the dosage form selected for (**a**) children with a chronic condition and (**b**) for healthy children.

When focusing on children that selected a medicine already used as their favourite (93% of respondents), there seemed to be a remarkable preference for liquid over other dosage forms in healthy children of age groups 0–6 years (76.8%) and 7–12 years (54%). Despite this trend declining with age increasing, liquid was still chosen by 21% of adolescents, as shown in Figure 5. On the other hand, the percentage of children with a chronic condition selecting liquid was less than 40% among the youngest (0–6 and 7–12 years) and decreased to 5% in the oldest age group. When looking at solid dosage forms, tablets were selected by less than 10% of healthy children between 7 and 12 years, while their selection was doubled in chronically ill children of the same age. Similarly, the figures for capsules were four and three times higher for children with a chronic disease of 0–6 years and 7–12 years, respectively, compared to the healthy ones. The trends for other dosage forms remained flat in the two younger groups of healthy children, whereas the choice selection of participants with a chronic condition was wider and independent of their age. The range of dosage forms selected by children with a long-term illness is more varied than that of the healthy ones, as children with a chronic condition are more likely to be exposed and experience different types of oral dosage forms.

Children with a long-term illness are obliged to take one or more medicines daily, and their inclination for solid dosage forms could be explained by the portability of these formulations and possibly fewer palatability issues. This speculation was confirmed by looking at the characteristics affecting the choice of their favourite medicine; the options: ‘it is easy to take’ and ‘it is quick to take’ appeared more relevant for those children with a chronic condition, taking medicines frequently, than the healthy ones.

**Figure 6:**
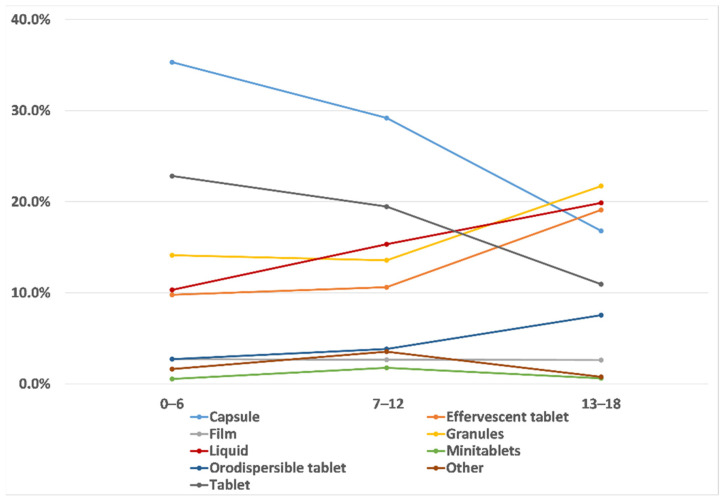
Least favourite medicines’ trends among the three age groups: 0–6 years, 7–12 years, and 13–18 years.

The analysis of their least favourite medicine showed that these were capsules and tablets for children between 0 and 12 years, while it was granules, liquid, and effervescent tablets for children between 13 and 18 years, as shown in Figure 6. In general, the aversion for capsules and tablets reduced significantly as age increased (R = −1.06 for capsules and R = −1.06 for tablets), whereas the aversion for granules and liquid significantly increased (R = 0.51 and R = 0.48, respectively) with the age of the children. Minitablets, orodispersible films, and orodispersible tablets were not usually selected, suggesting that children had few opinions for these dosage forms.

Interestingly, results showed a general attitude against granules, and this increased steadily with the age of the children. In all three age groups, the majority of those selecting granules never tried them before; thus, the choice was based mainly on their perception. Among those who selected granules as their least favourite medicine, the two characteristics that influenced their choice were taste and texture/mouthfeel, although interestingly, this was not based on real experience.

When looking at their health status, children with a chronic condition tended to dislike more liquid dosage forms than healthy children (23.5% vs. 15.7%, *p* = 0.0156). While healthy children had more aversion for solid dosage forms, such as capsules, granules, and tablets. Similarly, children taking medicines from time to time seemed to dislike more capsules and granules than those taking medicines regularly. This was in line with what was observed for their most favourite medicine.

**Figure 7:**
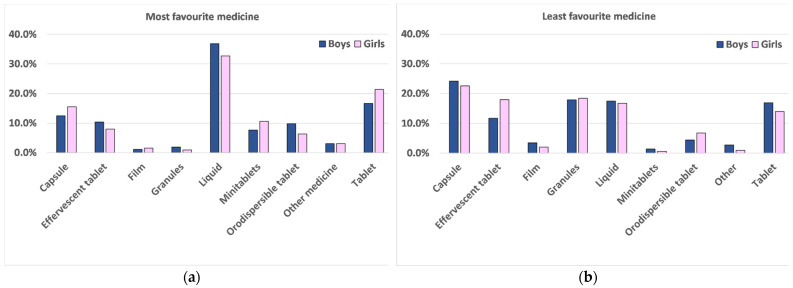
Percentage of boys (blue columns), and girls (pink columns) selecting (**a**) their most favourite and (**b**) their least favourite dosage form.

Finally, the analysis by sex showed that this did not have any significant influence in the selection of their most and least favourite medicine, indicating that boys and girls of the same age tend to have similar attitudes for oral dosage forms, Figure 7.

**Figure 8:**
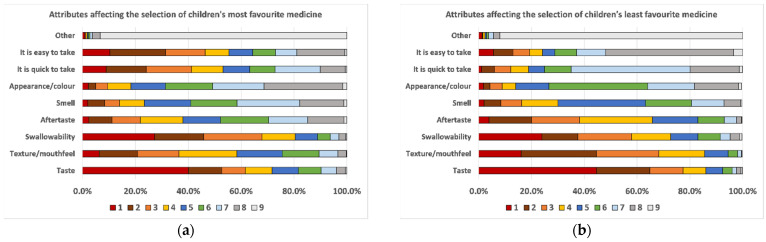
Dosage form attributes that affected the selection of children’s (**a**) most and (**b**) least favourite dosage form. Children were asked to rank the nine attributes (taste, texture/mouthfeel, swallowability, aftertaste, smell, appearance/colour/it is quick to take, it is easy to take, or other) by ordering them from the most relevant (number 1) to the least relevant (number 9).

Children were also asked to rank the attributes that affected the selection of their most and least favourite dosage form by ordering the nine items suggested from the most relevant (number 1) to the least relevant (number 9). Taste and swallowability (intended as the size of the solid or the amount of liquid taken) were the two most relevant attributes influencing the selection of both their most and least favourite medicine, Figure 8. In addition, the texture/mouthfeel was relevant for both selections, particularly for their least favourite medicine. The fact that medicine should be easy and quick to take seemed more relevant for the selection of their most favourite medicine, but not very relevant for their least favourite one, whereas aftertaste appeared more relevant for their least favourite medicine. The appearance/colour of the medicine and the smell seemed to be attributes of low relevance for defining children’s preferences.

Differences in the selection of attributes seemed to vary from a dosage form to another. For instance, as mentioned before, children who selected granules as their least favourite medicine mainly selected taste and texture/mouthfeel as the two most relevant features affecting their selection. On the other hand, for those children selecting capsules as their least favourite medicine, swallowability was the most relevant attribute, followed by taste, texture/mouthfeel, and their practicality (not easy to take). Taste was the major attribute affecting the selection of liquid formulations, whether it was chosen as the most or least favourite choice. Texture/mouthfeel and aftertaste were also relevant features, particularly for those children who considered liquid their least favourite medicine.

When dividing children according to their experience in terms of medicines use, few changes were observed in the selection of attributes between children who took one or more medicines on a daily basis and those who took medicines rarely. Swallowability and medicine practicality (it is easy and quick to take) seemed slightly more relevant for those taking medicines frequently, whereas taste for those taking medicines rarely. For their least favourite medicine, taste emerged to be the most important attribute for both groups, and no other relevant differences were observed.

## 4. Discussion

The collection of evidence-based data on formulation characteristics preferred by the children is crucial for understanding children’s opinions about dosage forms and for guiding the design of age-appropriate acceptable medicines. Regulators now underline the importance of children’s participation in research to understand their opinions about medicines [4]. Methods for involving children in research are still in development, and to allow their participation, it is important to create effective and age-appropriate tools that are compatible with their understanding and abilities [2,13]. In this study, an online survey created in collaboration with EPTRI PPI and YPAG groups and piloted with several children groups from different countries was used to involve the public in formulation advisory activities and to collect information that could facilitate the development of better paediatric dosage forms. The use of a survey distributed in collaboration with YPAG groups proved to be a relatively quick and easy inclusive method to reach a large and heterogeneous group and able to expand the role that children and young people should play in informing not only clinical but also basic and translational research. The study focused on oral dosage forms as this is the route most commonly used in paediatric patients [6], but the YPAG groups have proven to be a great asset to reach out end-users.

Direct experience with a dosage form was a major factor affecting children’s favourite choice, and most of the children (93%) in this study chose as their favourite a dosage form already used. Concomitant other factors affecting children’s preferences were frequency of medicine use, which was related to their health status, and the age of the children.

Moreover, results from this study highlighted that some attributes of dosage forms were more relevant than others in affecting children’s preferences. In particular, taste and swallowability were the main two attributes affecting respondents’ choice for both their most and least favourite medicine. An attribute more important for selecting their favourite dosage form was the practicality of a medicine (easy and quick to take), whereas texture/mouthfeel and aftertaste were relevant attributes for the selection of their least favourite medicine. On the other hand, the appearance/colour and smell of a medicine seemed to be attributes of low importance. Similar results were observed by Ranmal et al. in their study, where taste and size of dosage forms were rated as very important, whereas colour was ranked as the least important attribute [2]. Furthermore, our study underlined slight variations in the selection of attributes from a dosage form to another, whereas the frequency of medicine use seemed not to affect the selection of attributes.

Liquid emerged to be the most favourite dosage form overall. Most of the children who selected liquid were young or healthy and taking medicines rarely; the latter ones represented the majority of participants in this study, explaining why liquid dosage forms were widely selected. This could be because the range of dosage forms healthy children are exposed to is narrow or because medicines for non-chronic conditions such as fever or cough are still mainly available as liquid formulations. Moreover, these children might find it hard to swallow a solid dosage form compared to children with experience since they are not used nor trained to do that. A previous study evaluating the ability of children to swallow different-sized placebo tablets reported that 79% of participants unable to swallow at least one tablet, were in fact, ‘tablet naïve’ children [14].

In the past, liquid dosage forms were considered the most appropriate formulations for the paediatric population, as deemed easy to swallow without risk of choking and better for dose flexibility, catering for all age groups [15]. However, more than a decade ago, it was recognised that liquid dosage forms present several drawbacks over solid medicines, such as exposure to undesirable excipients, extra palatability, stability challenges, and higher costs. Already in 2008, the World Health Organization (WHO) was encouraging the use of flexible solid dosage forms as the preferred oral formulations for children, instead of liquid medicines [16,17].

On the other hand, sub-groups of children who had already experienced medicines in various dosage forms, such as some adolescents and children with a chronic disease, preferred solid dosage forms to liquid. Children with a chronic disease are persuaded and trained to take solid dosage forms from a relatively early age, and they tend to appreciate them due to their portability over liquid forms; they are easy to take in any environment (e.g., school) and practical when the administration is repeated frequently. Among the characteristics in support of their favourite choice, the options: ‘it is easy to take’ and ‘it is quick to take’ were more relevant for those children with a chronic condition than the healthy ones. Moreover, it should be noted that some children with a chronic condition take more than one medicine per day, and their preferences are usually related to the overall treatment plan they are following rather than a specific dosage form.

Respondents of this survey seemed not to be acquainted with novel flexible dosage forms such as orodispersible tablets and films. The main reason for their limited use and selection could be that without sufficient national market availability, accessibility and, therefore, use cannot be guaranteed. In addition, their cost is still higher compared to conventional solid dosage forms [15,18], and the fact that healthcare professionals still prefer or are accustomed to prescribing conventional dosage forms over the others in children could be added hurdles.

Specifically, orodispersible films were among the least selected options in terms of most and least favourite dosage forms, indicating a clear lack of knowledge for this dosage form. A previous study assessing the acceptability of orodispersible films in children found that 83% of caregivers were unfamiliar with this dosage form before the study. The few acceptability studies that focused on orodispersible films reported positive feedbacks from children and their caregivers [19,20]; nonetheless, orodispersible films are currently only used for niche clinical conditions.

From our analysis, a very small but positive attitude for these novel dosage forms emerged. Children who selected their preferences based on perception instead of experience (7% of the surveyed population) chose orodispersible films and orodispersible tablets as second and third choice in age group 0–6 years, and effervescent tablets, minitablets, and orodispersible tablets as their first three choices in age group 6–12 years. This indicates an attraction for these novel formulations and a shift of paradigm from the belief that all young children want liquid dosage forms. Overall, these findings seem to support formulators to develop more paediatric medicines in these types of dosage forms. Assessing key drivers and barriers to implementation would be needed to understand how to encourage healthcare professionals and caregivers to support the prescription, administration, and use of these dosage forms over liquid.

However, a negative attitude for granules emerged, especially among adolescents. Although the use of granules emerged not to be widespread in the population surveyed, they were the second least favourite dosage form selected, clearly indicating that children have a negative opinion about granules. Reasons might be that granules are believed to be formulations for the youngest children or not practical or unpleasant to take since they are frequently mixed with food or liquids. Previous studies have reported that multiparticulates such as granules have palatability issues due to their grittiness perception in the mouth [21], and results from the current study concordantly reported that texture/mouthfeel and taste were the two most relevant attributes affecting their selection as least favourite medicine. The reasons for these selections are worth to be explored further, and it should raise awareness among formulators to carefully study how to enhance palatability to improve acceptability of these dosage forms. In addition, these negative feelings could be reduced by doing more educational activities about granules to familiarise children and carers with this type of oral dosage form.

In the past, the main concern impeding the use of solid dosage forms in children, particularly in infants and toddlers, was the risk of choking. However, several studies focusing on minitablets demonstrated the ability of young children to take these dosage forms safely [22,23,24,25]. Klingmann et al. demonstrated that the administration of multiple minitablets was feasible, well-tolerated, even better than syrup, and safe for all children 6 months and older, shifting the paradigm from liquid to solid dosage forms once and for all [26].

After increasing confirmation that children can swallow small solid dosage forms, the focus of paediatric formulation scientists have now shifted into conventional, yet small, dosage forms, i.e., tablets, to find the optimal features to reach acceptable, age-adapted dosage forms so to ‘normalise’ their use in the paediatric population. In their feasibility study, Bracken et al. [14] evaluated the acceptability of different-sized placebo tablets (6 mm, 8 mm, and 10 mm) in children aged 4–12 years. Before their study, the empirical knowledge about the optimal tablet size for children of different ages was inadequate. Their results highlighted that the majority of children who attempted to swallow tablets successfully did so, including healthy children. The 8 mm tablets were deemed the most acceptable size by all participants, concluding that tablets are an acceptable formulation for children aged 4–12 years. A larger definitive trial is now expected to confirm what was observed in this study [14].

A better understanding of the optimum dimensions for paediatric tablets across age groups, as well as the influence of physical and sensory aspects (such as shape, surface coating, or bitterness), is highly valuable for patient-centred medicine development [27]. A recent study proved that the bitterness of tablets does not seem to affect the ability to swallow them, but the sensory properties determine whether the tablets are acceptable or not [28].

Several strategies for learning how to swallow solid dosage forms have been studied, including behavioural approaches and head position training [29], most of them with positive outcomes. When taught how to take solid dosage forms, children as younger as 5 years have proved to be able to learn quickly how to do that. The conduct of activities involving healthcare professionals, parents, and children, such as the KidzMed project [30], is a useful strategy able to introduce children to tablets and help them to familiarise and take these dosage forms [30].

Research into age-appropriate, acceptable, solid dosage forms is growing and with positive results. However, from our analysis, it emerges that the transition from liquid to solid and flexible dosage forms has not reached the general paediatric community yet. More work and pill swallowing training are required in order to shift the negative ‘perception’ of solid dosage forms, e.g., tablets, especially in younger age groups and healthy children.

## 5. Strengths and Limitations

To our knowledge, this was the first study to look at children’s opinions for oral dosage forms encompassing all age groups and across several European countries.

It is known that acceptability of different routes of administration varies among different countries in Europe and other nations, and similarly acceptability for different oral dosage forms [6]. Cultural and religious factors may affect palatability and formulation preferences of end-users; however, studies focusing on these characteristics are still very few. There are no studies yet that have looked at differences in oral dosage form preferences across Europe. Our results suggest that some national variations in dosage form preferences exist. Noteworthy, in Spain, liquid dosage forms emerged to be largely used and liked by the entire paediatric population; in the Netherlands and Belgium, minitablets are widely used and appreciated in particular by adolescents. These variations in preferences across countries could be related to different market availability of dosage forms as well as different cultural preferences of healthcare professionals, caregivers, or end-users that directly or indirectly might affect the regional availability of dosage forms or the other way round. Another speculation is that educational campaigns to support the uptake of specific dosage forms performed in some countries might have increased the visibility and usage of some dosage forms over others. These variabilities are important to know to allow companies to better meet the needs of certain market subsets. It would be interesting to conduct further investigation in this direction involving more European countries to obtain a more representative sample of European children and to understand if there are real patterns of national differences in dosage form preferences.

A limitation of this study was that in each country, the number of incomplete or not submitted surveys was large, and for two countries (Sweden and Czech Republic) the number of surveys completed was deemed not sufficient to be included in our analysis. The nature of the survey and the type of channels used for dissemination did not allow to follow up participants and to understand the underlying causes that led several people to leave the survey before the end. This could be because they found the questions difficult to understand or the survey too long, although language and layouts were deemed appropriate by the YPAG groups. Understanding the reasons beyond that would be essential to improve the completion rate in the future. Furthermore, the completion rate over time revealed that in each country, the majority of responses occurred in the few months after the launch of the survey and plummeted after that. This indicates the importance of conducting follow-up actions to keep getting responses over longer periods of time. Interestingly, before publishing the survey in the UK and Netherlands/Belgium, the question order was slightly changed, allowing the child to immediately start the survey if allowed by the parent. This change increased the completion rate, with 0% of incomplete surveys submitted in both countries. This shows that even the structure of the survey can affect people’s adherence.

Secondly, it was not possible to check the quality and reliability of the responses (e.g., the actual age of the respondents). This is a common limitation of survey-based studies, where people may not feel encouraged to provide accurate or honest answers. Whilst there is the possibility that a participant might have given inaccurate answers, we expect this to be limited to a small percentage of the population surveyed. We used the commitment of participants to complete the survey as an arbitrary parameter for response reliability. Hence, only the responses to those questionnaires that were entirely completed were included in this analysis. However, this might have added a selection bias as respondents who completed the survey might have been children more interested in medical research compared to the whole population. This is a challenge associated with all voluntary surveys. On the other hand, the advantages associated with this methodology are that it can be easily administered to potential respondents living in different geographical regions, enabling them to reach out to a wide number of participants from a large number of countries. An in-loco study of the same scale would be costly and impractical to conduct.

Moreover, the majority of children taking the survey were healthy and taking medicines sporadically. This could be because the number of children affected by a chronic illness is smaller compared to the healthy ones and because the channels used to disseminate the survey might have led to this imbalance, e.g., more schools instead of hospitals or paediatric research networks.

Furthermore, children did not try the dosage forms they were asked to choose from. Thus, they only relied on their personal opinions and preferences to select their most and least favourite dosage form. This could be considered a limitation because children in this study have different experiences with oral medications, with the majority of them being exposed to a few of them. This might have introduced bias in their selections as they only considered those formulations they already knew, ignoring all the others, and it may not necessarily reflect behaviours of the same children having to take these medicines. However, these results capture the general attitudes and factors that can affect children’s acceptability in real-life conditions, and they show a picture of the current situation in terms of market availability and use of oral dosage forms in several EU countries.

## 6. Conclusions

This study provided interesting insights about children’s perceived preferences for oral dosage forms in a diverse European sample of children and showed the feasibility and value of using an online survey to reach the general paediatric population.

Results highlighted that children’s attitudes for a dosage form are influenced by factors such as age, experience with certain dosage forms, health status, and the frequency of medicine use, whereas sex did not influence preferences. Moreover, taste and swallowability emerged to be important attributes for the selection of their most and least favourite medicine. Liquid was the most used and favourite dosage form overall. This was widely selected by children less than 12 years old and by those without any chronic condition that in this study represented the majority of participants. Monolithic solid dosage forms, i.e., tablets and capsules, were mostly chosen by adolescents and by children of any age with a chronic health condition taking medicines frequently. Less conventional dosage forms, i.e., orodispersible films and tablets, minitablets, and granules, were not usually used nor selected; among them, granules emerged to be the least appreciated.

National variations worthy of note were the large use and preference of liquid formulations by the Spanish participants and the positive attitude towards minitablets of the Dutch participants.

Although these results do not represent dogmatic guidance, they provide further insights into determinants affecting medicine preferences to keep in mind when designing tailored paediatric dosage forms and to understand the general attitudes of the paediatric population.

## Figures and Tables

**Table 1:** Timeframe (number of months) during which the survey circulated in each country, and the types of dissemination channels used to distribute it.

Country	No. Months	Dissemination Channels
Spain	15	Individual emails to several schools collaborating with KIDS Barcelona Members of the KIDS Barcelona YPAG and friends Social media
Italy	14	EPTRI, CVBF, and TEDDY websites Social media (LinkedIn, Facebook, Twitter, WhatsApp, and Instagram) Newsletters Monthly meetings and round tables of the KIDS Bari members Website of some high schools of the KIDS Bari members By email to all the members of the paediatric hospital in Bari To medical doctors, physiologists, teachers, and nurses participating in the KIDS Bari training activities and initiatives Through friends and parents of the KIDS Bari group
Albania	14	Websites of schools of KIDS Albania members Social media (Facebook, WhatsApp, Instagram, and LinkedIn)
Romania	12	Social media (Facebook) Romanian Stop TB Partnership
United Kingdom	10	Generation R Young Person’s Advisory Group Alliance made up of approximately 17 YPAGs across the UK Social media Parents’ organisations and groups
The Netherlands	5	PedMED ‘Kind en Onderzoek’ website Communication channels of the VSOP By email to the Dutch-speaking sites of the Belgian Paediatric Clinical Research Network (BPCRN) Social media (Twitter)
Belgium	5	

**Table 2:** Number of questionnaires submitted, fully completed (second column), and partially completed (third column) by countries.

Country	Complete Questionnaires Submitted	Incomplete Questionnaires Submitted
Spain	267	425
Italy	407	401
Albania	172	29
Romania	135	14
United Kingdom	89	0
Czech Republic ^1^	-	14
Sweden ^1^	-	1
Netherlands and Belgium	102	0
Total	1172	884

^1^ Countries excluded from the analysis due to a low response rate.

**Table 3:** Demographics of participants in each country.

	United Kingdom	Spain	Romania	Albania	Italy	Netherlands and Belgium	Total
Respondents (%)	89 (7.6)	267 (22.8)	135 (11.5)	172 (14.7)	407 (34.7)	102 (8.7)	1172
Age							
0–6 (%) 7–12 (%) 13–18 (%)	16 (18.1) 32 (35.9) 41 (46.0)	56 (21.0) 98 (36.7) 113 (42.3)	48 (35.5) 71 (52.6) 16 (11.9)	18 (10.4) 31 (18.0) 123 (71.6)	22 (5.4) 59 (14.5) 326 (80.1)	24 (23.5) 48 (47.1) 30 (29.4)	184 (15.7) 339 (28.9) 649 (55.4)
Sex							
Boys (%) Girls (%)	39 (43.8) 50 (56.2)	127 (47.6) 140 (52.4)	62 (46.0) 73 (54.0)	56 (32.6) 116 (67.4)	189 (46.4) 218 (53.6)	48 (47.0) 54 (53.0)	521 (44.0) 651 (56.0)
Chronic disease							
No (%) Yes (%)	57 (64.0) 32 (36.0)	229 (85.8) 38 (14.2)	120 (88.9) 15 (11.1)	151 (87.8) 21 (12.2)	371 (91.2) 36 (8.8)	44 (43.1) 58 (56.9)	972 (83.0) 200 (17.0)

## Data Availability

Not applicable.

## Supplementary Materials

The following are available online at https://www.mdpi.com/article/10.3390/pharmaceutics13050730/s1.
