# Supplementary material for: Children’s Preferences for Oral Dosage Forms and Their Involvement in Formulation Research via EPTRI (European Paediatric Translational Research Infrastructure)
Source: Pharmaceutics. 2021 May 15;13(5):730. doi: 10.3390/pharmaceutics13050730 (PMC8156390; doi:10.3390/pharmaceutics13050730)
Supplement: Supplementary file 1 [file pharmaceutics-13-00730-s001.zip › pharmaceutics-1184298-supplementary.pdf]

# Questionnaire

- ○ Option 1: I am a child and will be doing this survey myself. – Start from question 6.
- ○ Option 2: I am the adult helping a child to complete this survey and happy to do so.

Q1 Age of the adult

18 …110

Q2 Gender of the adult

- ○ M
- ○ F

Q3 Relationship with child

- ○ Parent
- ○ Grandparent
- ○ Sibling
- ○ Other relative
- ○ Other

Q4 Highest education level achieved

- ○ No school
- ○ Primary School
- ○ Secondary School
- ○ Higher education

From now on, you should be answering the questions.

Q6 How old are you (years)?

Less than 1, 1…18

Q7 What is your gender?

- ◯ Boy
- ◯ Girl

Q9 Make up a name for yourself ________________________________________

Q10 In which Country do you live in?

Afghanistan… Zimbabwe

Q11 Where were you born?

Afghanistan… Zimbabwe

Q12 Choose one or more ethnic group that you consider yourself to be:

- ▢ White
- ▢ Black/African/Caribbean
- ▢ Chinese
- ▢ Indian/Pakistani/Bangladeshi
- ▢ Other Asian
- ▢ Arab
- ▢ Other

Q13 If you selected "other" from the previous question, could you please specify your ethnic group below?

________________________________________________________________

Q14 Do you have a chronic illness? (this is a problem that lasts over three months or more)

- ◯ Yes
- ◯ No

Q15 Do you take medicines at home?

- ◯ Many times daily
- ◯ Once daily
- ◯ From time to time

Q16 Can you select your **MOST FAVOURITE** type of medicine from the list below?

- ◯ Capsule
- ◯ Tablet
- ◯ Liquid
- ◯ Orodispersible tablet
- ◯ Effervescent tablet
- ◯ Minitablets
- ◯ Film
- ◯ Granules
- ◯ Other medicine not in the list above but that you take orally (please tell us more below)

Q17 If you chose "other medicine" from the previous question, please describe it below

- ◯ Other medicine_____________________________________________

Q18 Tell us more about your MOST FAVOURITE: have you taken it before?

- ◯ I am taking it at the moment
- ◯ I have taken it in the past
- ◯ I have never taken it

Q19 What do you like the most about your MOST FAVOURITE? Please order the items below from the most relevant to the least relevant
______ Taste
______ Texture/mouthfeel
______ Swallowability/size of solid/ amount of liquid
______ Aftertaste
______ Smell
______ Appearance/colour
______ It is quick to take
______ It is easy to take
______ Other

Q20 If you selected "Other", please specify________________________________________________

Q21 If you have taken this medicine before, have you felt any discomfort while taking it for the first time?

- ○ Yes
- ○ No

Q22 If so, has the discomfort continued after the first time you took the medicine?

- ○ Yes
- ○ No

Q23 Can you select your **LEAST FAVOURITE** type of medicine from the list below?

- ○ Capsule
- ○ Tablet
- ○ Liquid
- ○ Orodispersible tablet
- ○ Effervescent tablet
- ○ Minitablets
- ○ Film
- ○ Granules
- ○ Other medicine not in the list above but that you take orally (please tell us more below)

Q24 If you chose "other medicine" from the previous question, please describe it below

○ Other medicine ____________________________________________

Q25 Now tell us about your LEAST FAVOURITE: have you taken it before?

○ I am taking it at the moment

○ I have taken it in the past

○ I have never taken it

Q26 What do you not like about your LEAST FAVOURITE? Please order the items below from the most relevant to the least relevant

______ Taste
______ Texture/mouthfeel
______ Swallowability/size of solid/ amount of liquid
______ Aftertaste
______ Smell
______ Appearance/colour
______ It takes time to prepare/take it
______ It is complicated to prepare/take
______ Other

Q27 If you selected "Other", please specify ______________________

Q28 If you have taken this medicine before, have you felt any discomfort while taking it for the first time?

○ Yes

○ No

Q29 If so, has the discomfort continued after the first time you took the medicine?

○ Yes

○ No

Q30 Are you happy with your answers? If so, click on the blue arrow below. But before you do it, can you tell us how you did this quiz?

○ Yourself

○ With a little help from your grownup

○ With a lot of help from your grownup
